# Time-resolved neurotransmitter detection in mouse brain tissue using an artificial intelligence-nanogap

**DOI:** 10.1038/s41598-020-68236-3

**Published:** 2020-07-09

**Authors:** Yuki Komoto, Takahito Ohshiro, Takeshi Yoshida, Etsuko Tarusawa, Takeshi Yagi, Takashi Washio, Masateru Taniguchi

**Affiliations:** 1 The Institute of Scientific and Industrial Research, Osaka University, 8-1 Mihogaoka, Ibaraki, Osaka 567-0047 Japan; 2 Artificial Intelligence Research Center, The Institute of Scientific and Industrial Research, Osaka University, 8-1 Mihogaoka, Ibaraki, Osaka 567-0047 Japan; 3 KOKORO-Biology, Laboratories for Integrated Biology, Graduate School of Frontier Biosciences, Osaka University, 1-3 Yamadaoka, Suita, Osaka 565-0871 Japan

**Keywords:** Nanobiotechnology, Bionanoelectronics, Sensors

## Abstract

The analysis of neurotransmitters in the brain helps to understand brain functions and diagnose Parkinson’s disease. Pharmacological inhibition experiments, electrophysiological measurement of action potentials, and mass analysers have been applied for this purpose; however, these techniques do not allow direct neurotransmitter detection with good temporal resolution by using nanometre-sized electrodes. Hence, we developed a method for direct observation of a single neurotransmitter molecule with a gap width of ≤ 1 nm and on the millisecond time scale. It consists of measuring the tunnelling current that flows through a single-molecule by using nanogap electrodes and machine learning analysis. Using this method, we identified dopamine, serotonin, and norepinephrine neurotransmitters with high accuracy at the single-molecule level. The analysis of the mouse striatum and cerebral cortex revealed the order of concentration of the three neurotransmitters. Our method will be developed to investigate the neurotransmitter distribution in the brain with good temporal resolution.

## Introduction

**Figure 1:**
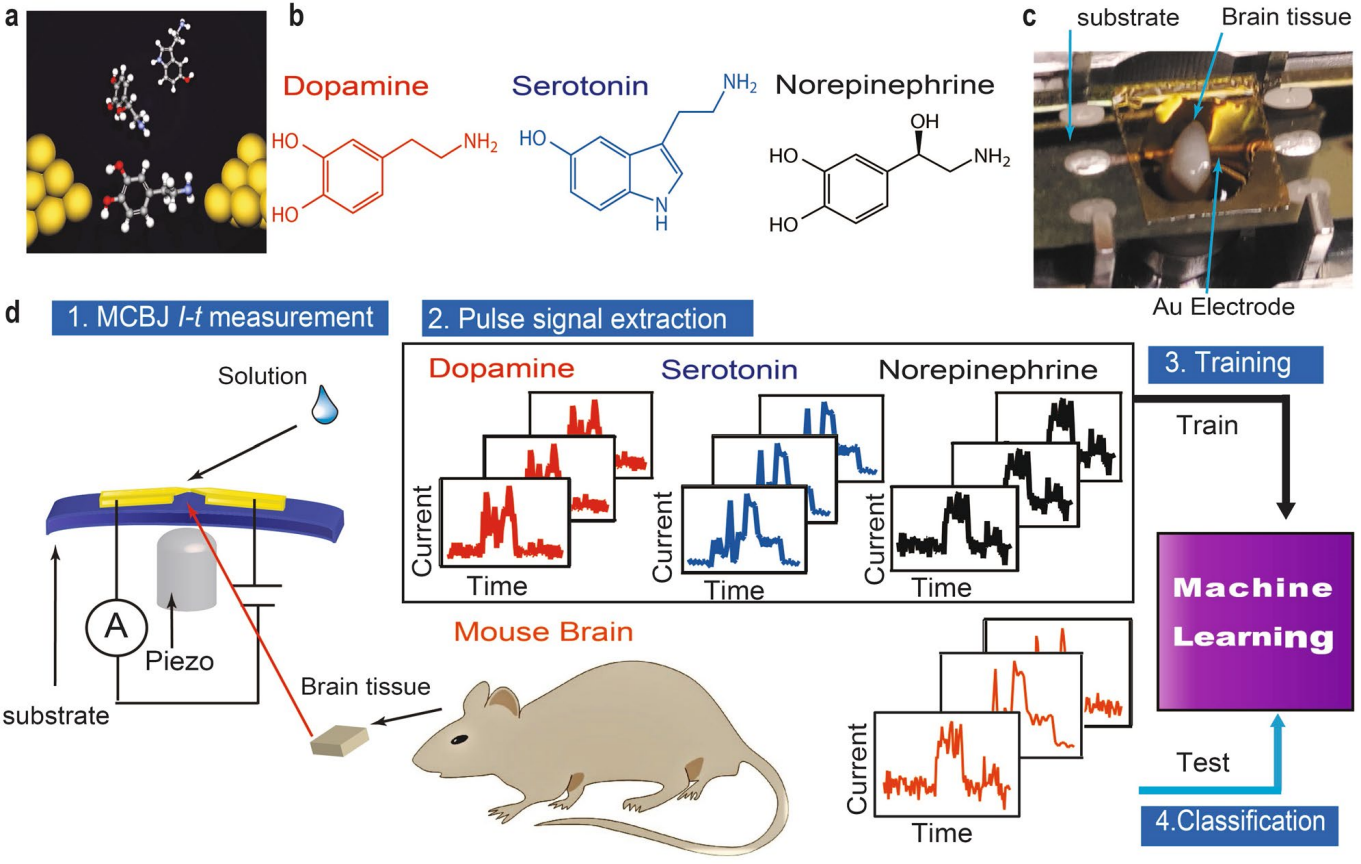
Detection of single neurotransmitter molecules. (**a)** Schematic of single-molecule detection. (**b)** Molecular structures of dopamine, serotonin, and norepinephrine. (**c)** Experimental setup for mouse brain analysis; brain tissue mounted onto a mechanically controllable break junction (MCBJ) substrate using narrow gold wires (as electrodes) drawn via electron beam lithography and covered with SiO_2_. (**d)** Analysis flow: the neurotransmitter solutions are dropped onto MCBJ substrates for training, the mouse brain tissue is then mounted onto MCBJ substrates and the current measurements are performed while these substrates are bent using piezo to form nanogaps. The pulse signals are classified via supervised machine learning.

Neurotransmitters travel through synaptic clefts and transmit nerve impulses in the brain. Investigation of their role and the nerve action is essential to understanding consciousness, motivation, memorisation, and some diseases^1–4^. Studies about the reward system used to control incentive salience have been mostly focused on dopamine (DA)^1,2^. Some recent reports have suggested that the reward system is connected by the interplay between dopamine and serotonin (5-HT) or norepinephrine (NE)^5–8^. Moreover, the lack of DA in Parkinson’s disease is well known^9,10^. The contribution of other neurotransmitters, such as 5-HT and NE, is also attracting attention^9,11^. The role of neurotransmitters is generally analysed using pharmacological inhibition experiments^5^; microdialysis, voltammetry^12^, and mass spectrometers^13,14^; and optical detection^15,16^. However, these methods are insufficient to understand neurotransmitter interplay in the brain due to the lack of temporal resolution, In sharp contrast, there is a technique based on nanoelectrodes for electrically detecting and distinguishing single molecules with good temporal resolution in a nanometre-sized gap^17–27^. In the mechanically controllable break junction (MCBJ) method, one of the single-molecule measurement methods, nanogaps are formed by breaking a metal nanowire. The conductance of a single molecule between a nanogap is measured. Single molecule measurements using MCBJ were initiated to investigate the basis of conduction in simple molecules such as BDT and molecular hydrogen^17,18^. Following the development of single-molecule measurement techniques, the main purpose of a single-molecule measurement was the development of molecular devices such as single-molecule diodes^19,20^ and transistors^21–23^. In recent years, single-molecule measurements techniques such as DNA, RNA sequencing have been reported^23–27^. Since single-molecule measurements directly measure the conductance of molecules between the gaps, multiple types of neurotransmitters can be detected simultaneously in a single nanogap without any chemical modifications. However, there is a plurality of target neurotransmitters and other molecules in the brain and conventional single-molecule measurement methods can hardly identify the target molecules selectively and accurately in such conditions because single-molecule conductance is easily affected by molecular orientation, contact geometries, and contamination. Improvement of the discrimination method in single-molecule measurement is effective for investigating neurotransmitter interplay. To solve this problem, we developed an analysis method based on machine learning (ML) from the waveform of the tunnelling current flowing between nanoelectrodes via a single-molecule^28^. ML enables higher accuracy identification of multiple types of target molecules (e.g., neurotransmitters in the brain) in the presence of noise. In this study, we measured the relative amount of the three neurotransmitters, dopamine, norepinephrine, and serotonin, in different parts of the mouse brain (Fig. 1).

## Results and discussion

**Figure 2:**
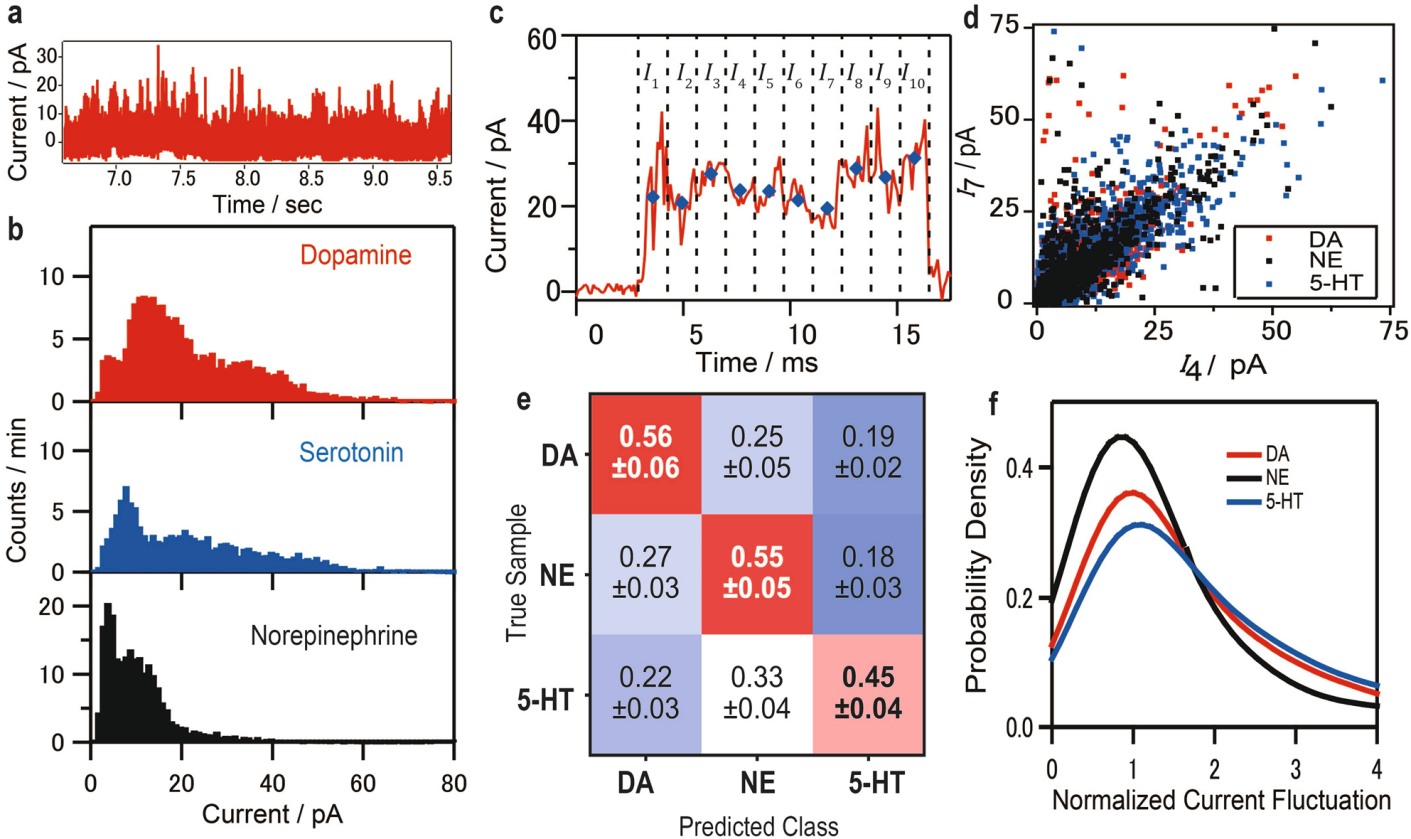
Current measurements of single neurotransmitter molecules and classification results via a machine learning-based method. (**a)** Typical current–time profile of a dopamine aqueous solution via single-molecule measurement; the individual pulse signals were extracted as shown in the Supplementary Information. (**b)** Typical current histograms of dopamine, serotonin and norepinephrine, obtained under a bias voltage of 100 mV. The bin size is 1 pA. (**c)** Typical single-molecule signal of dopamine; the red curve and blue dots represent the current–time profile and the signal features, respectively (i.e., the average current values *I*_1_, *I*_2_, *I*_3_, *I*_4_, *I*_5_, *I*_6_, *I*_7_, *I*_8_, *I*_9,_ and *I*_10_, which were 22.1, 20.8, 27.6, 23.7, 23.5, 21.4, 19.5, 28.8, 26.8, and 31.3 pA, respectively). The black dotted lines divide the pulse signals into 10 regions along the time axis. (**d)** Typical feature currents (*I*_4_ and *I*_7_) of the single-molecule signals of dopamine (DA), norepinephrine (NE), and serotonin (5-HT). (**e)** Confusion matrix of the machine learning classification; the ratios and errors represent the average and standard deviation values of 10 classification results. (**f)** Probability density of the current fluctuation factors of DA, NE, and 5-HT.

Training and classification of the pure single neurotransmitter signals are necessary for brain measurement. Figure 2a shows part of the current trace obtained via single-molecule analysis of a DA solution. Signals for NE and 5-HT were also obtained and are shown in Figure S2 in SI3. The current traces of 5-HT show large fluctuations. The NE conductance is smaller, and a current profile difference seems to exist between the neurotransmitters. However, the conductance of a single-molecule junction can be easily changed. Therefore, a stochastic approach is necessary for the analysis of single-molecule measurements. The current histograms of DA, 5-HT, and NE are displayed in Fig. 2b. Slight differences are observed in the histograms. However, the large overlap between the histograms and noise signals hinders the discrimination of the origin of the pulse signals (SI 4). Their respective averaged currents are 15 ± 7, 15 ± 7, and 14 ± 7 pA. The similarity observed in the histogram shapes suggests the necessity of ML.

The ML algorithm used in this study required the conversion from signals to feature vectors^20^. We adopted ten-dimensional feature vectors of ten average current values from the corresponding signal regions along the time axis, as shown in Fig. 2c for a typical current pulse signal in the case of DA single-molecule measurements. As expected from the current histograms, Fig. 2d reveals similar distributions among the three neurotransmitters. However, slight differences were observed; for example, serotonin was common where the difference between the fourth and seventh current was large.

The single neurotransmitter signals after noise removal^28,29^ were classified via supervised ML using the XGBoost classifier^30^. We used the data with a gap width of 0.56 nm because the detection rates of the three neurotransmitters are the same (see Figure S5c). Figure 2e illustrates the confusion matrices of the three neurotransmitters, consisting of the average results of a tenfold cross-validation; in all cases, the accurate classification ratios exceeded the random classification ones. We successfully classified the neurotransmitters, which were not distinguishable from the current histograms. The F-measure—an index representing the classification performance—was 0.52, which is higher than the value for random classification, namely, 0.33. The false prediction caused by the large overlap in the histograms is due to the similar chemical structures of the neurotransmitters rather than because of the limited number of current data or intrinsic noise of the MCBJ (SI 6 and Figure S8). However, a classification F-measure of 0.52 is selective enough for its practical application because the accumulation of signals improves the selectivity. In our method, each signal is classified one by one; the molecule is classified using the majority vote of all signal classification results. For instance, we consider the case in which the prediction ratio of the true sample is 0.5, whereas those of the others are 0.3 and 0.2 (see SI 5 and Figure S9). The accuracy obtained using the majority vote is 80% for 20 signals, 90% for 40 signals, and 99% for 110 signals. Hence, the F-measure of 0.52, shown in Fig. 2e, is sufficient for discriminating the single signals of the three target molecules using stochastic analysis. As an alternative to the XGBoost classifier, we also used a random forest classifier, achieving an *F*-measure of 0.50 (see Figure S11 in SI 8). The essence of the discrimination seemed to not be the classifier but the current measurement and features. We developed single neurotransmitter molecule classifiers by training the ML algorithm using single-molecule signals from neurotransmitters and blank measurements, with our noise removal method.

Next, we evaluated how ML improves the accuracy of discriminating between neurotransmitter molecules which have similar conductance histograms. Figure 2f shows the probability density, obtained via kernel density estimation, of the signal current fluctuations. Here, the current fluctuation is defined the difference between the maximum current in 10 separate regions and the average current normalised by the average current. The results indicate that the single-molecule current changed most easily in the order of 5-HT, DA, and NE. The single-molecule current depends on the structure of the molecular junction^31,32^. The current fluctuation order is related to the occurrence of conformational changes of junctions resulting from molecular structures, such as the size of π planes^33–35^ and intramolecular interactions (SI 9)^36^.

**Figure 3:**
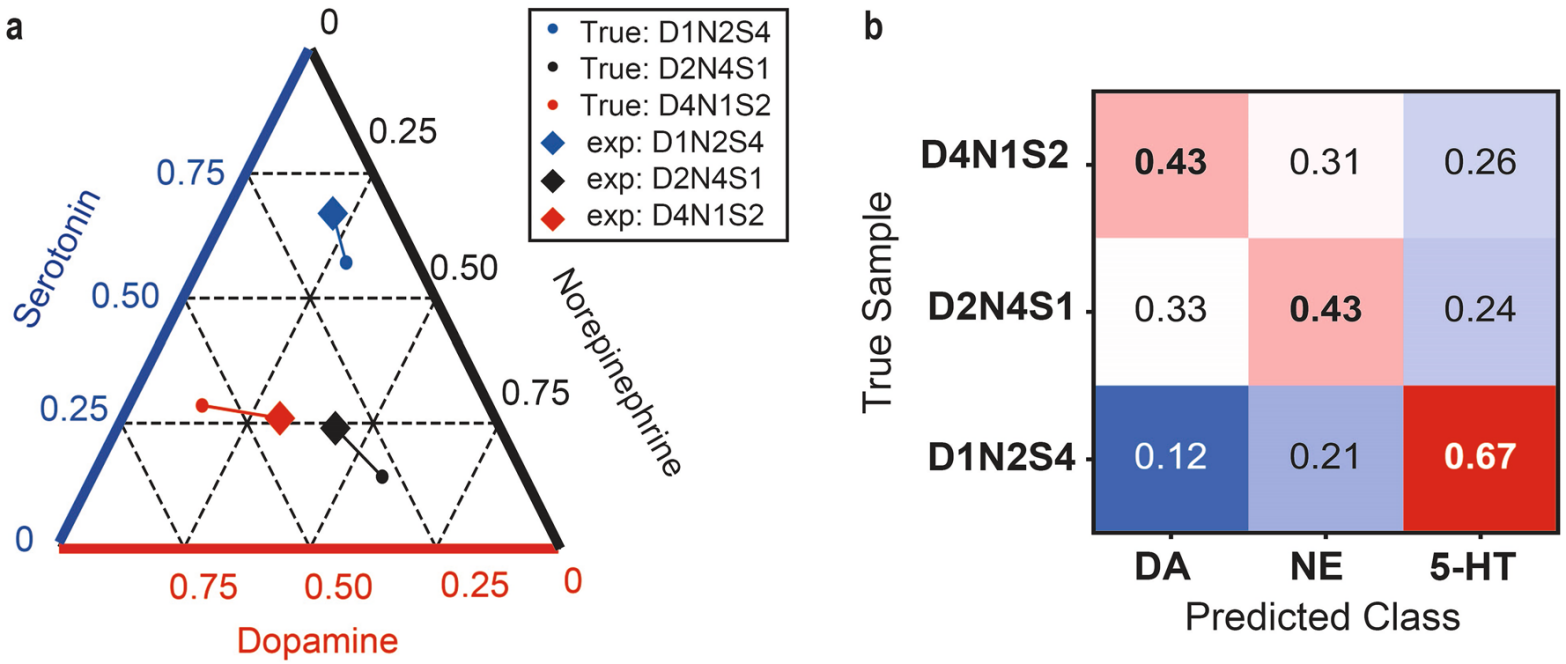
Machine learning classification of neurotransmitter mixtures with three different ratios of dopamine (DA), norepinephrine (NE), and serotonin (5-HT), namely 1:2:4 (D1N2S4), 2:4:1 (D2N4S1), and 4:1:2 (D4N1S2). (**a)** Ternary plot of the classification results; the small dots and diamonds represent the true and predicted mixture ratios. For instance, the blue dot denotes true mixture ratio of DA:NE:5-HT = 1:2:4 (0.14:0.28:0.57), and the blue diamond denotes the ratio of the predicted label counts of D1N2S4; DA:NE:5-HT = 0.12, 0.21, 0.67. (**b)** Ratio of classification result of mixtures of monoamine neurotransmitters. Each row represents a mixture sample. Each column represents the ratio of the signal counts predicted for each neurotransmitter using ML classification.

Mixtures of the three neurotransmitters were also analysed to confirm the proposed discrimination method using ML. First, the classifier was trained with signals from each neurotransmitter solution; then, it was used to classify the signals from the mixtures with different DA:NE:5-HT ratios, namely, 1:2:4 (D1N2S4), 2:4:1 (D2N4S1), and 4:1:2 (D4N1S2), respectively. The experimental mixing ratio indicates the ratio between the counts of each target molecule. The classification results are illustrated in Fig. 3. In the ternary plot (Fig. 3a) of the three neurotransmitters, each classification ratio was closest to the true concentration ratio of the corresponding mixture. The ratios of the classification results shown in Fig. 3b reveal that the molecules with the highest concentration are correctly predicted. Although the neurotransmitters were still not quantitatively detected in this experiment, the proposed method allowed us to distinguish the labelled data.

**Figure 4:**
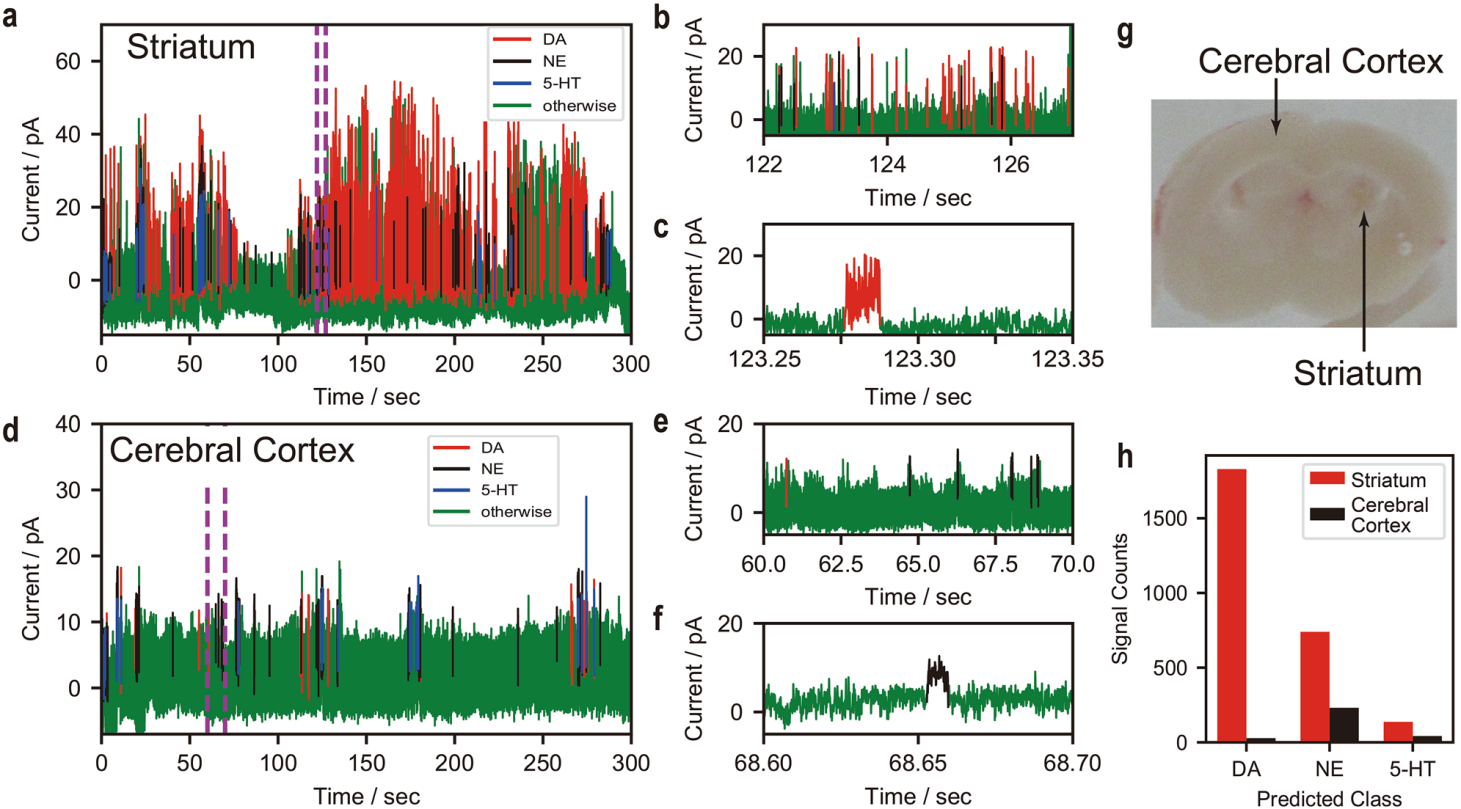
Classification of the pulse signals of mouse brain tissues. (**a**–**f)** Current signals of dopamine (DA), norepinephrine (NE), and serotonin (5-HT), classified via machine learning, along with noise signals from the striatum (**a**–**c**) and cerebral cortex (**d**–**f**); the plots on the right show zoomed in images of the current profile portions, as identified by the dotted lines, in (**a**,**d**). (**g)** Mouse brain slice; the slices were separated into 1-mm sections for measurements. (**h**) Classified signal counts from the striatum and cerebral cortex; the measurement time was 300 s.

After the development and validation of an ML classifier for neurotransmitter detection in solutions, we finally demonstrated the detection of monoamine neurotransmitters in mouse brain tissues (striatum and cerebral cortex) via the proposed method. For the single-molecule measurements, both the brain tissue samples and the nanogap electrodes were immersed in artificial cerebrospinal fluid (ACSF). ACSF maintains the neuronal activity in brain slices. Figure 4a–f shows the current–time profiles for the striatum and cerebral cortex samples shown in Fig. 4g; we could detect the pulse signals with a time resolution of 10 ms. The current signals included various noise signals from other neurotransmitters, contaminants and the migration of the gold electrodes. These noise signals were removed using a positive and unlabelled data (PU) classifier^28,29^, a suitable algorithm for distinguishing between two classes when only positively labelled and unlabelled data are given. The detailed analysis procedure is described in the Supplementary Information. In this study, we performed this noise removal twice before classification with the ML classifier; the first round was conducted to obtain neurotransmitter signals as training data for the second round and supervised ML classification (see SI 11). The relationship between the detection rate after noise removal and gap width suggests the validity of our noise removal method. Noise signals caused migration of electrodes observed even to be blank in the first PU classification step. Biological samples, such as brain tissues contain various contaminants, non-targeted molecules, whose signals are removed by the second PU classification step. The counts of the classified signals for DA, NE, and 5-HT were, respectively, 1829, 740, and 137 in the striatum samples and 25, 231, and 42 in the cerebellar cortex sample as shown in Fig. 4h. The higher concentration of DA in the striatum sample was revealed using nanogap measurements and ML analysis; the good qualitative agreement with previous methods using liquid chromatography/tandem mass spectrometry demonstrates the effectiveness of the proposed method for the detection of monoamine neurotransmitters^37,38^. The stochastic current dwell time analysis is shown in Figure S15 in SI. The large overlap between the neurotransmitters implies that our ML-based analysis method is effective for discrimination of the neurotransmitters. In this experiment, our method detected the neurotransmitters diffused into the ACSF. Our study shows that monoamine neurotransmitters are detected selectively even in contaminated samples through a combination of a single-molecule measurement and an ML-based analysis.

## Conclusion

In summary, monoamine neurotransmitters in the mouse brain can be detected with single-molecule measurements using our ML method that enables discrimination of even mild current profile differences due to the molecular structures. Our study also demonstrated the applicability of single-molecule measurements to various contaminated biological samples with high temporal resolutions. Moreover, we found that integration of multiple nanogap electrodes on a single chip is possible via semiconductor technology. We plan to develop our method further in order to investigate neurotransmitter distribution in the brain with high spatial and temporal resolutions. Improvements in the spatial resolution and in vivo noise endurability of our method combining a single-molecule measurement and an ML-based analysis will facilitate us to study and discover more complex nerve systems.

## Methods

### Preparation of neurotransmitter solutions

Dopamine hydrochloride and DL-norepinephrine hydrochloride were purchased from Sigma Aldrich Co. Ltd. Serotonin hydrochloride was purchased from Tokyo Chemical Industry Co., Ltd. The sample molecules were used without further purification. The neurotransmitter samples were dissolved in MilliQ water. The concentration of the solutions was 1 μM. In the mixed sample solutions, the neurotransmitters were present at molecular ratios of DA:NE:5-HT of 1:2:4 (D1N2S4), 2:4:1 (D2N4S1), and 4:1:2 (D4N1S2), and the minimum neurotransmitter concentration was 1 μM.

### Mouse brain preparation

All experimental procedures were in accordance with the Guide for the Care and Use of Laboratory Animals of the Science Council of Japan and were approved by the Animal Experiment Committee of Osaka University. The brain for the experiments was removed from a sacrificed 10-week-old female C57BL/6J mouse and sliced using a vibratome and razor. The size of the slice was 1 mm^3^. The electrical measurements were performed on the brain tissue samples mounted on MCBJ substrates and immersed in ACSF.

### Device fabrication

First, a polyimide film was coated as an insulating layer onto a thin-silicon substrate with spin coating. A gold nanowire was formed on the substrate using electron-beam lithography. Then, an SiO_2_ film was coated on the gold nanowire using chemical vapor deposition. The narrowest part of the nanowire is 100 nm. Dry-etching was performed to remove the polyimide layer under the gold nanowire and form a free-standing gold-wire.

### Electrical measurements

Single-molecule conductance measurements were performed at the optimal gap distance of the nanogap electrodes as described previously^24–26^. A lithographically fabricated gold nanowire on a thin silicone substrate was broken by mechanically bending the substrate under ambient condition with application of a bias voltage of 100 mV, and the single detection part of the nanogap electrodes was formed. Throughout the junction breaking process, the junction conductance (*G*) was monitored using a picoammeter (Keithley 6487). A series of conductance jumps of the order of *G*_0_ = 2*e*^2^/*h* (where *e* and *h* are the elementary charge and Planck’s constant, respectively) was observed, and the final conductance was 1 *G*_0_. Several seconds after reaching the 1 *G*_0_ state, a gold atomic junction naturally ruptured in the nanowire, creating a nanogap. The gap size was controlled using the piezo bias voltage. The gap distance was estimated from the baseline tunnelling current (see SI 1). The current profiles were recorded with a sampling rate of 10 kHz using a home-made 10^9^ A/V amplifier and digital multimeter (National Instruments PXIe-4081). After the measurements, the nanoelectrodes were approached and the metal junction was reformed by retracting the piezo for solution measurement. The substrates were then washed with Milli-Q water and the next sample solution and another solution were dropped onto them. Data for different solutions were obtained on one substrate to remove differences between substrates. For the mouse-brain experiments, the substrates were used only once.

## Supplementary information

Supplementary information.

## Data Availability

The datasets obtained and/or analysed during the current study, and computer code are available from the corresponding author on reasonable request.
