# Supplementary material for: Time-resolved neurotransmitter detection in mouse brain tissue using an artificial intelligence-nanogap
Source: Sci Rep. 2020 Jul 9;10:11244. doi: 10.1038/s41598-020-68236-3 (PMC7347941; doi:10.1038/s41598-020-68236-3)
Supplement: Supplementary file 1 — Supplementary information. [file 41598_2020_68236_MOESM1_ESM.pdf]

Supplementary Information for

Time-resolved neurotransmitter detection in mouse brain tissue using an artificial intelligence-nanogap

Y. Komoto, T. Ohshiro, T. Yoshida, E. Tarusawa, T. Yagi, T. Washio, & M. Taniguchi*

Correspondence to: taniguti@sanken.osaka-u.ac.jp

This PDF file includes:

SI1. Schematic structure of MCBJ substrate

SI2. Estimation of gap distance

SI3. Single-molecule signals for the neurotransmitters

SI4. Single-molecule prediction based on conventional histogram-based analysis

SI5. Detail of noise removal with PUC method

SI6. Detail of Supervised ML

SI7. Improvement of classification accuracy by accumulation

SI8. Classification results with different classification algorithm

SI9. Origin of classification

SI10. Analysis for signals from mixture

SI11. Analysis of mouse brain signals

SI12. Current and Dwell time histograms of mouse brain signals

Reference

SI1. Schematic structure of MCBJ substrate

Fabrication process is described in method section in main manuscript.

A Au narrow wire is fabricated on elastic substrate. Polyimide insulating layer is removed by dry-etching. Narrowest part of the Au wire is free-standing structure. The substrate was bended by pushing using piezo from backside (see Figure 1d in main manuscript). The nanogap was formed by breaking Au wire by bending. The nanogap width is controlled by piezo displacement. Estimation of gap width is described in next section in this supporting information.

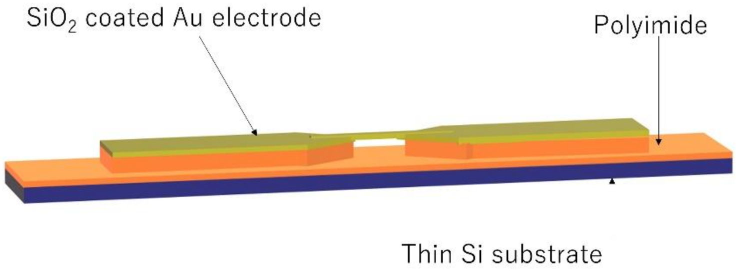

Figure S1. Schematic structure of MCBJ substrate

SI2. Estimation of gap distance

The gap distance is estimated using by following current equation of direct tunneling current

$$I = const\,\exp\left(-\frac{4\pi}{h}\sqrt{2mwl}\right).$$

Here, h, m,w, and l represents plank constant, electron mass, work function of gold electrode, gap distance. We used electron mass of 9.1×10^{-31} kg as m, and work function of Au(111), 5.3 eV as w. Effective mass and work function of gold nanogap not (111) surface should be used for accurate estimation. Furthermore, the inelastic gold gap broadening just after breaking atomic junction is not under consideration. Hence, the experimental gap length is underestimated. This underestimation provides disagreement between experimental gap distance and molecular size.

SI3. Single-molecule signals for the neurotransmitters

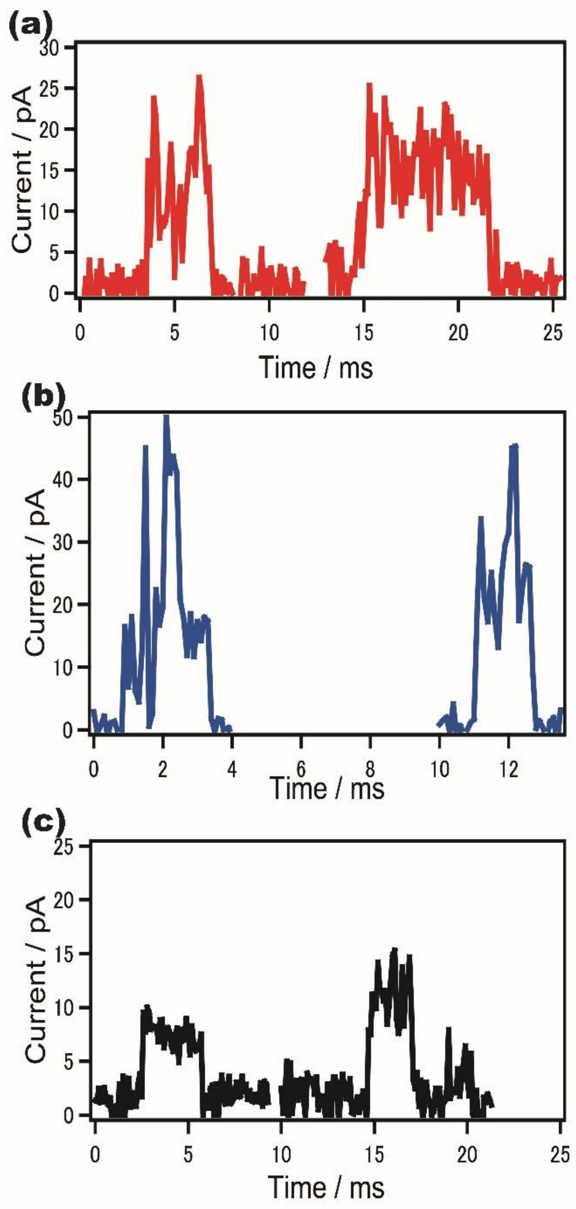

Figure S2. Examples of pulse signals from current measurements. a Dopamine. b Serotonin. c Norepinephrine.

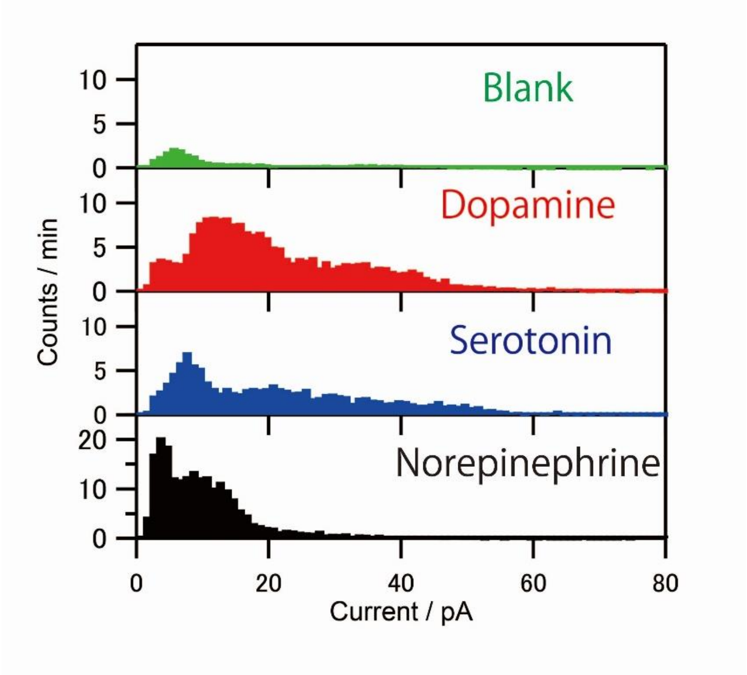

Figure S3. Current histograms of single-molecule neurotransmitters. The current was measured under a bias voltage of 100 mV.

SI4. Single-molecule prediction based on conventional histogram-based analysis

The signals were analyzed by the conventional histogram-based method, where only single features, such as the maximum and average currents, are analyzed to identify single-molecule signals. In this section, we only use the maximum current for signal discrimination. The current histograms per time unit are regarded as a probability density function for the molecules. Hence, the single-molecule signals are well-discriminated by comparing the detection rate in each current region, that is, by choosing the maximum probability densities among the target molecules when the overlap between the histograms of those molecules is small. The classification results obtained by the conventional histogram-based detection rate-comparison method are presented in Figure S4. Figure S4a shows the classification results for signals from pure solutions while Figure S4b shows the classification of signals obtained from mixtures. Figures S4a and S4b correspond to Figures 2e and 3b, respectively. Both Figures S4a and S4b show a wrong discrimination. The conventional histogram-based analysis does not provide an accurate discrimination between the three monoamine neurotransmitters because the overlapping of the conductance histograms of the neurotransmitters is large.

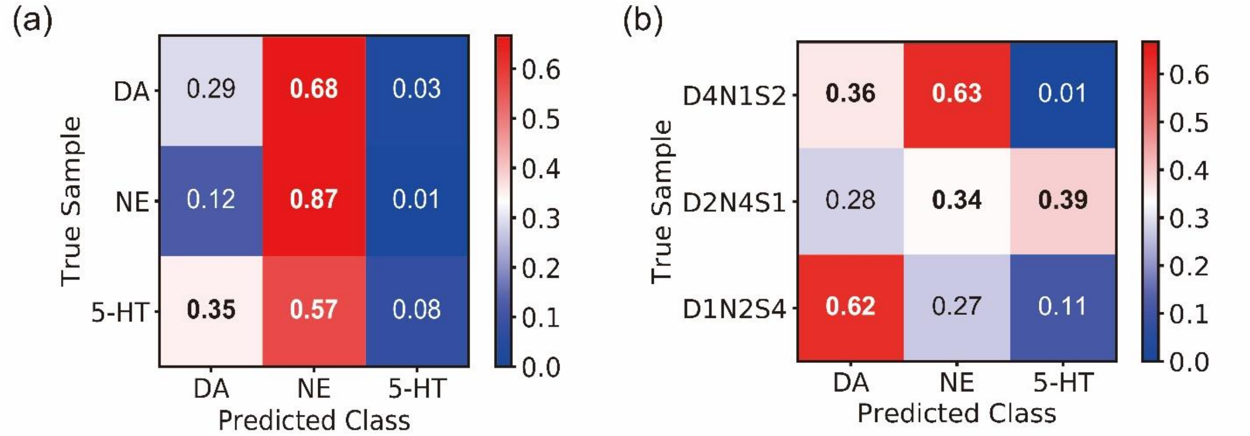

Figure S4 Classification result by conventional histogram-based comparison. classification results for signals from pure solutions (a) and from mixtures (b). The classification is performed by choosing a neurotransmitter which shows highest histogram count regarded as probability densities in the signal current region.

SI5. Detail of Noise removal with PUC method

To remove noise signals, we perform Positive and Unlabelled data Classification (PUC). Schematic image of PUC is shown in Figure S5a. PUC is appropriate algorithm for noise removal. Some signals are observed even in blank solution (See Figure S3, reference *28* in main text.). At first, to remove the blank signals, we performed PUC as shown in Figure S5b, and Fig. S5c represents the relation between gap width and signal count per unit time as recognised obtained via PU classification. The detection rates of dopamine and norepinephrine are similar and both decrease with gap broadening. This is due to the associated decrease in current since the coupling between molecule and electrode weakens so that the molecules cannot be detected as signals even if passing through the gap. The detection rate of serotonin behaves differently; it does not decrease with the gap broadening from 0.56 to 0.58, and 0.60 nm. Dopamine and norepinephrine consist of a benzene ring, while serotonin contains an indole ring and is larger than them. Thus, serotonin is detectable in larger gaps. The detection rate of the signals obtained with the PU classifier can be explained by the molecular structures. This result implies the possibility of screening specific neurotransmitters due to control by the gap width. The molecular size is not in agreement with the gap width, estimated from the tunnelling current. The effective mass and the accurate work function of the gold nanoelectrodes are out of consideration. The underestimation of the gap width leads to a discrepancy between experimental gap distance and molecular size. Furthermore, we performed supervised ML without PUC noise removal as shown in Figure S6. DA signals are not predicted correctly. From this result, PUC is effective for noise removal.

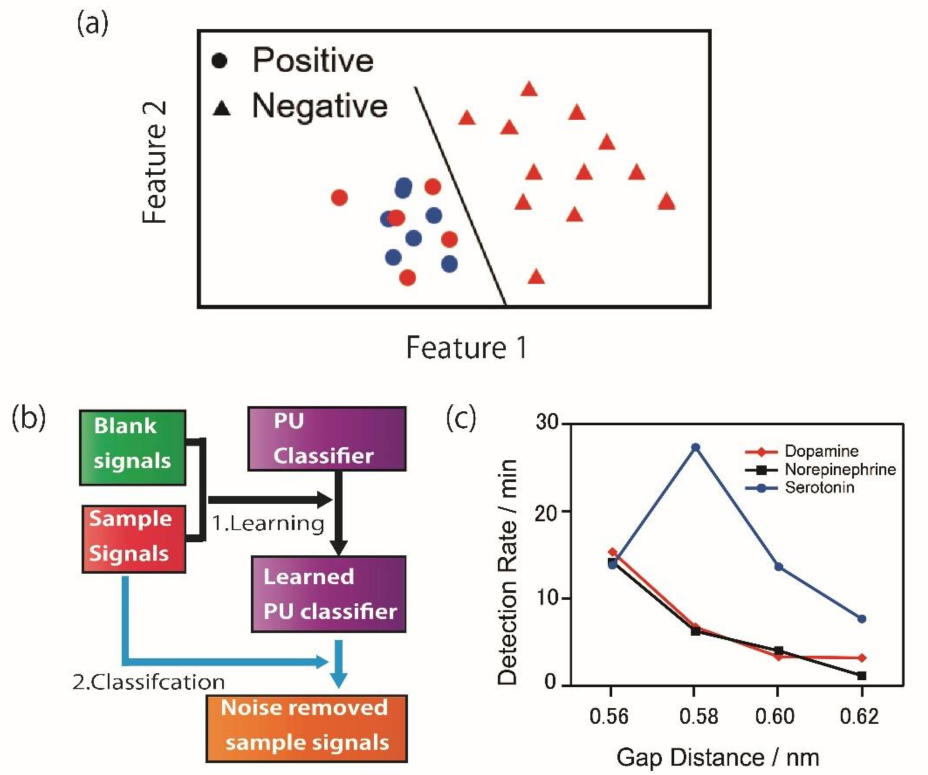

Figure S5. Positive and unlabelled data (PU) classifier, developed by Elkan and Noto. a Schematic PU classification: the blue colour represents the positive data, which contain only one class, and the red colour indicates the unlabelled data, consisting of both the positive and negative classes. The class of unlabelled data was unknown before PU classification; the PU classifier determines the decision surface of these positive and unlabelled datasets. b Schematic flow of the noise removal method based on the PU Classifier. c Relation between gap distance and detection rate of single-molecule neurotransmitter signals after noise removal.

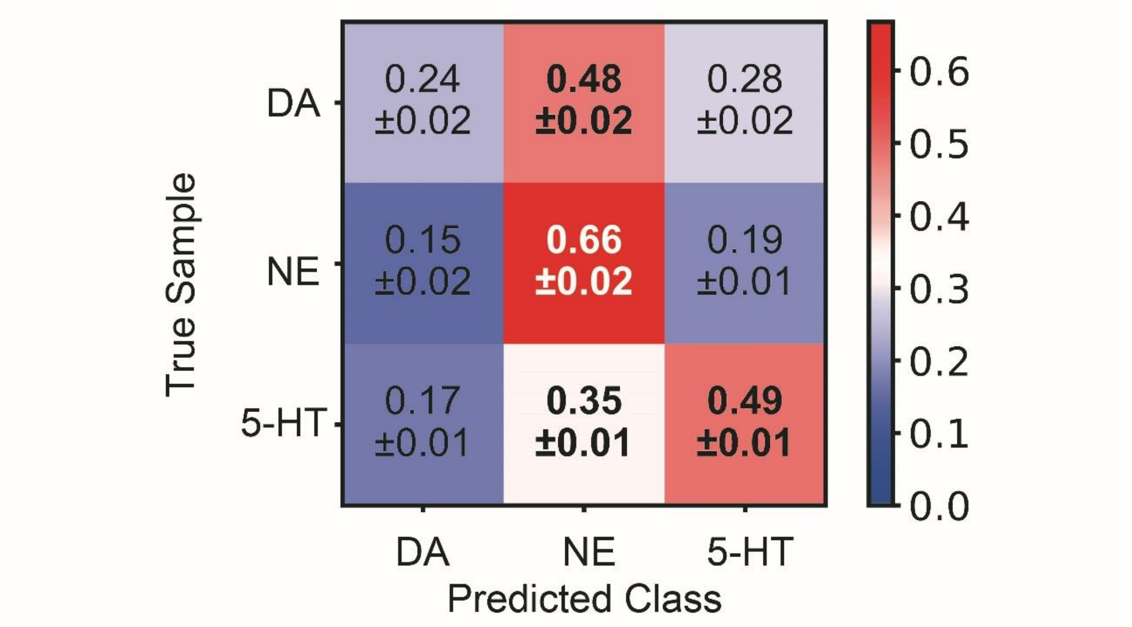

Figure S6. Confusion matrix of classification without noise removal. Each 6000 signal was used. Dopamine (DA) signals were predicted as norepinephrine (NE) with the highest ratio. This result and Fig. 2e demonstrate the effectiveness of the positive and unlabelled data classification noise removal method.

SI6. Detail of Supervised ML

Each 1000 neurotransmitter signals recognised via the positive and unlabelled data classifier-based noise removal method were used for the training and tested via machine learning by the XGBoost classifier with default parameters. A 10-fold cross-validation was performed and its average and standard deviation values provided the classification ratios and errors.

The classification was evaluated based on the weighted *F*-measure average. The *F*-measure was 1 for a complete classification and 0.33 for a random classification of three classes; it is defined as the harmonic mean of two trade-off performance indicators, recall and precision, which are expressed as

$$\text{Recall} = \frac{TP}{TP + FN}$$

and

$$\text{Precision} = \frac{TP}{TP + FP}$$

where TP, FN, and FP denote true positive, false negative, and false positive, respectively.(See Figure S7.)

The analysis was performed using Python 3.6 with the XGBoost library. We also analysed with random forest classifier from scikit-learn library version 0.21.1 instead of XGBoost classifier. The classification result with random forest is represented in SI.8.

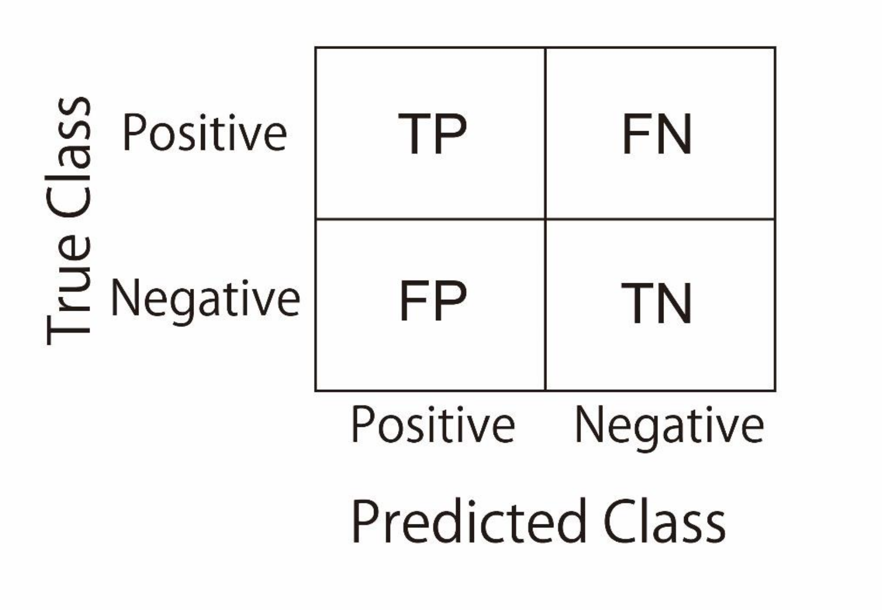

Figure S7. Confusion matrix to define the classification results.

Figure S8 shows the relation between the number of signals for training data and the F-measure of the classification. The F-measure saturates at about 500 signals, which demonstrates that the false detection is not caused by a limited number of current data. The intrinsic noise of the MCBJ method does not affect the accuracy either. The F-measure of classification among four DNA monomers, obtained by the MCBJ method, is 0.83, which is higher than the value determined for the neurotransmitters (see Ref. 28 of the manuscript). False prediction is due to similar molecular structures.

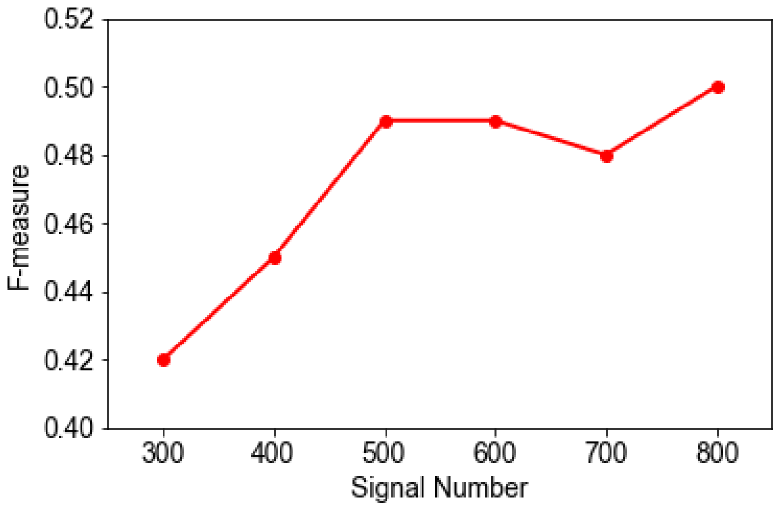

Figure S8 Relation between number of signals for training data and classification F-measure.

SI7. Improvement of classification accuracy by accumulation

The classification performance index (F-measure) for discrimination between the three neurotransmitters is 0.52, which is slightly higher than the value for random classification. This accuracy is not the accuracy determined by using multiple signals during application but only that for a single pulse. The classification accuracy can be improved by statistical analysis. In the method reported in this manuscript, each signal is classified one by one; the molecule is classified with majority vote of all signal classification results.

Here, we consider the relation between the classification accuracy and the number of signals n. The prediction ratio for a single pulse of the true molecule p_1 is set to 0.5 while the prediction ratios of the other molecules p_2 and p_3 are set to 0.3 and 0.2, respectively. Then, the probability of accurate prediction by the majority vote P is described using the following equation:

$$P = \sum_{S} p_1^{k_1} p_2^{k_2} p_3^{k_3} \frac{n!}{k_1!\,k_2!\,k_3!}. \quad (1)$$

where k_i denotes the number of signals predicted as molecule I, S is the set of k values that satisfy: $k_1 > k_2 + k_3$. The relation between P and n is shown in Figure S9. The accuracy determined by the majority vote is 80% for 20 signals, 90% for 40 signals, and 99% for 110 signals.

The relation between the classification accuracy and the number of signals for the ratio represented in Figure 2e of the manuscript is shown in Figure S9. The accuracies for all the three neurotransmitter molecules are improved by accumulation, as shown in Figure S10.

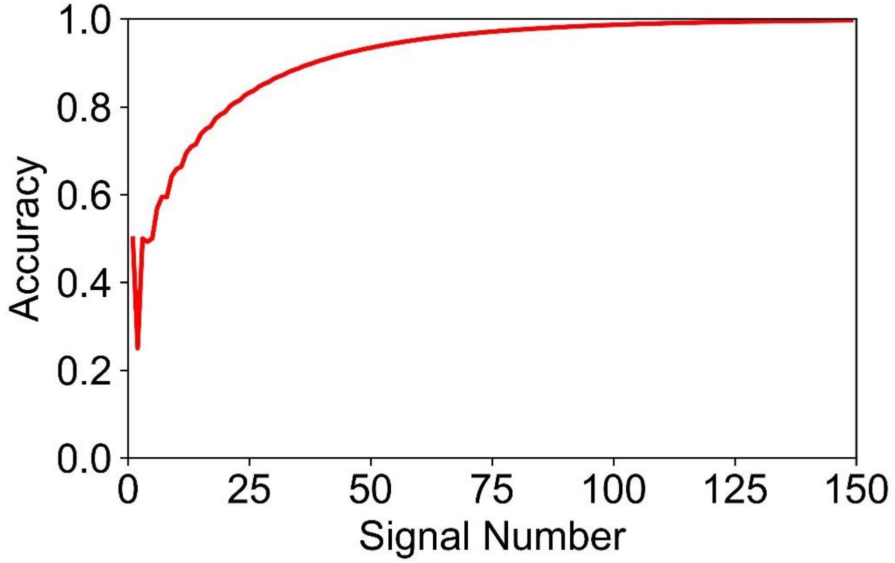

Figure S9. Relation between probability of accurate prediction by majority vote and number of signals. Prediction probability is 0.5, 0.3 and 0.2.

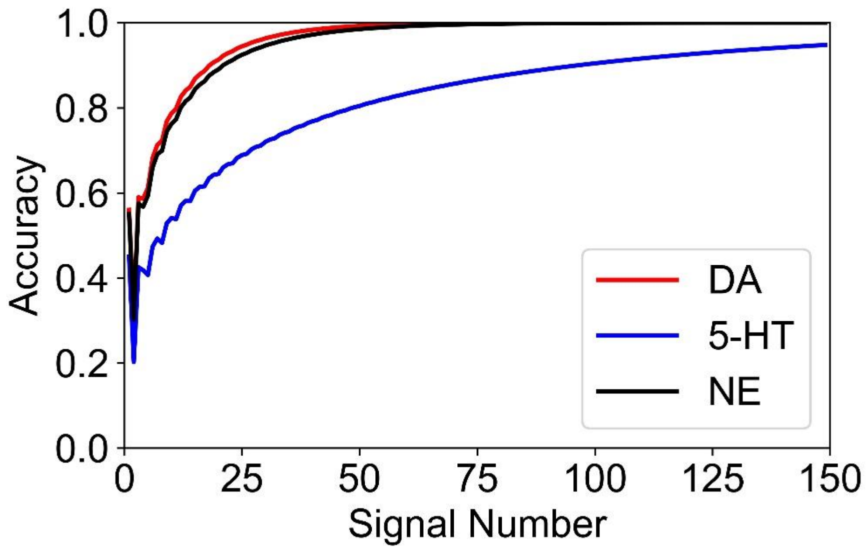

Figure S10. Relation between probability of accurate prediction of the three neurotransmitters and number of signals for the prediction probability in classification result.

SI8. Classification results with different classification algorithm

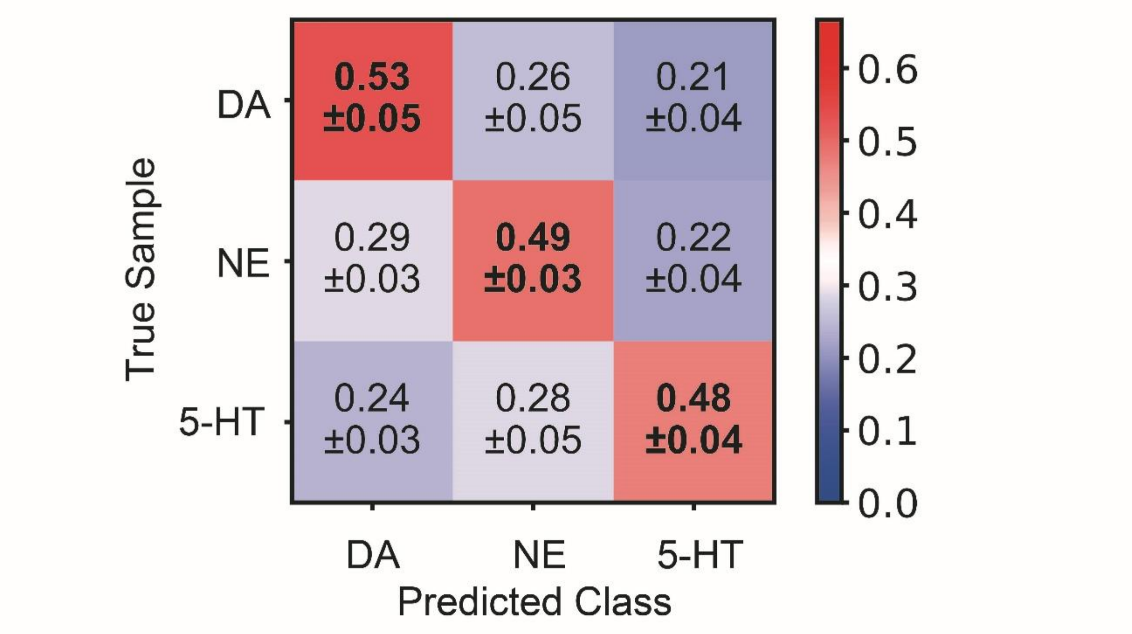

Figure S11. Confusion matrix classified by a random forest classifier. The confusion matrix is represented in Extended Data Fig. 3. The classifier in Scikit-learn ver. 0.20.2 was used.

SI9. Origin of classification

Discrimination between two neurotransmitters were also performed (Figure S12). All three case show accurate classification result. We compared serotonin and dopamine. The amino group is a typical anchoring group of single-molecule junctions and adsorbs onto gold electrodes via coordination bonds [39]. In absence of strong anchoring groups such as thiols, amino groups and π-conjugation planes act as anchoring groups [33,40-42]. Given the junction structure of serotonin and dopamine, both these molecules form bonds via the amino groups with one electrode and via the π-conjugation planes with the other one; they have the same amino groups but different π-conjugation planes. Since the indole ring of serotonin is larger than the catechol ring of dopamine, serotonin consisted from larger π plane is deduced to form various structures in the junctions that originate larger current fluctuations. Dopamine and norepinephrine differ only by one hydroxyl group in the molecular structure. Despite this slight difference, the spectroscopic results in the gas phase indicate that the dopamine conformers are more abundant than the norepinephrine ones because of the intra-molecular interactions due to the hydroxyl groups [36,43-45]. Conformational changes of the alkyl groups cause conductance changes [31,32], hence, the current fluctuation of norepinephrine is smaller than that of dopamine due to intra-molecular interactions. Although the current fluctuation factor was not directly used as a classification feature, the classification results suggest that the machine learning-based method classifies the molecules based on their behaviour in the nanogaps due to difference in the molecular structures. Analysis via machine learning could allow the observation of intra-molecular interactions at the single-molecule level.

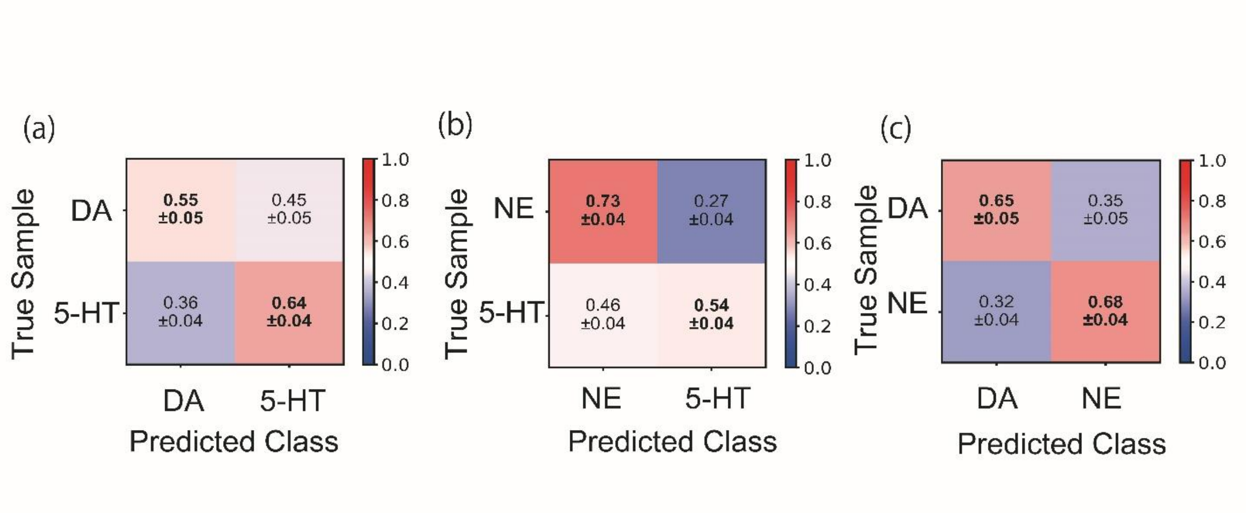

Figure S12. Confusion matrices of classification for two coexisting neurotransmitters. Dopamine, serotonin and norepinephrine are labelled as DA, 5-HT, and NE, respectively.

SI10. Analysis for signals from mixture

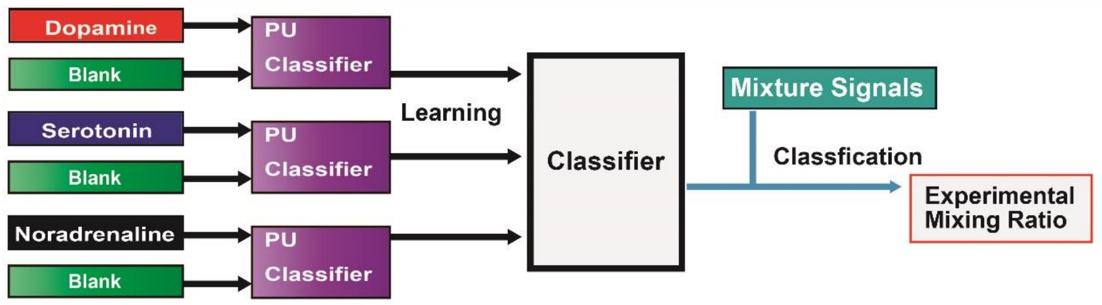

Figure. S13. Schematic flow for the classification of neurotransmitter mixtures. The machine learning classifier was trained with each neurotransmitter signals recognised via the positive and unlabelled data classification; the trained classifier discriminated all the signals from the mixtures, after noise removal. The experimental mixing ratios were derived from the ratios of signal counts for each predicted class.

SI11. Analysis of mouse brain signals

The detail scheme of mouse brain analysis is represented in Figure S14. In this scheme, we perform PUC twice, first PUC is noise removal for the noise signals observed in blank solution due to migration of gold electrodes or contamination. Second PUC is extraction of target neurotransmitters from contamination. There are many other molecules in mouse brain. The signals from first PUC only contain neurotransmitters signals. The noise removed signals are trained as positive data, we obtain targeted-neurotransmitters signals from contamination. Then neurotransmitters signals in mouse brain were classified with supervised ML.

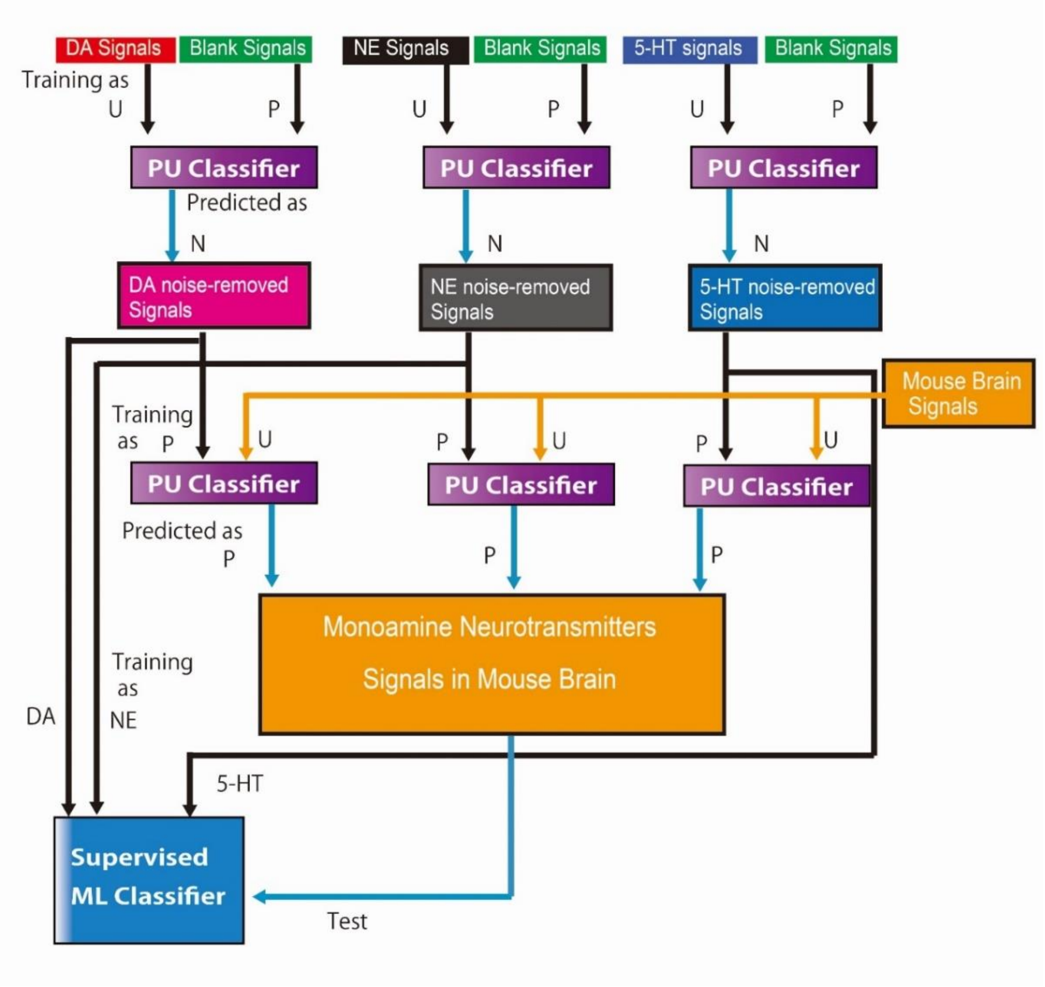

Figure S14. Schematic flow for mouse brain analysis. In the first positive and unlabelled data (PU) classification run, the PU classifier was trained using the signals from the neurotransmitter solutions as unlabelled data and those from the blank measurements as positive data. Then, the signals from the neurotransmitter solutions were recognised again. The signals classified as negative were adopted as single neurotransmitter signals for the next training data. The second PU classification run was performed to remove the noise signals originated from contamination in the mouse brain; for the training data, the signals obtained from the brain measurements were treated as unlabelled and those from the first PU classification run were considered as positive for each neurotransmitter. The elimination of the predicted negative signals for all the three neurotransmitters provided the neurotransmitter signals in the brain.

SI12. Current and Dwell time histograms of mouse brain signals

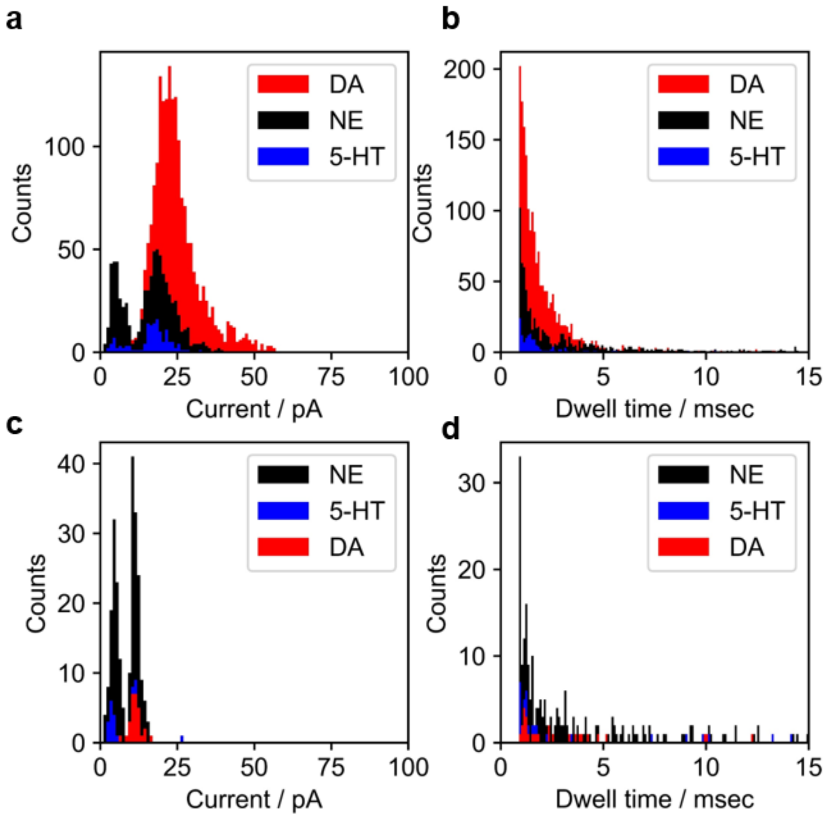

Figure S15. Current and dwell time histograms of mouse brain signals classified by ML analysis. a. Current histogram of classified signals of striatum. b. Dwell time histogram of classified signals of striatum. c. Current histogram of classified signals of cerebral cortex. d. Dwell time histogram of classified signals of cerebral cortex. Bin size is 1 pA for current histograms (a, c) and 0.1 msec for dwell time histograms (b,d) Colours indicates neurotransmitters predicted by ML analysis. Red, Blue, and Black represents by DA, 5-HT, and NE, respectively.

Current and dwell time histograms of mouse brain signals are shown in Figure S15. Compared striatum and cerebral cortex, striatum shows higher current signals caused by DA frequently.

However, focused on difference between the neurotransmitters, distributions of current and dwell time resemble each other. The neurotransmitters discrimination using current or dwell time histogram is difficult due to the similarity of histogram shapes. It suggests that ML-based analysis method is effective for neurotransmitters discrimination.

Reference

39. Venkataraman, L.; Klare, J. E.; Tam, I. W.; Nuckolls, C.; Hybertsen, M. S.; Steigerwald, M. L., Single-molecule circuits with well-defined molecular conductance. *Nano Lett.* **6**, 458-462 (2006).

40. Komoto, Y.; Fujii, S.; Kiguchi, M., Single-molecule junctions of π molecules. *Mat. Chem. Front.* **2**, 214-218 (2018).

41. Martin, C. A.; Ding, D.; Sørensen, J. K.; Bjørnholm, T.; van Ruitenbeek, J. M.; van der Zant, H. S., Fullerene-based anchoring groups for molecular electronics. *Nano Lett.* **130**, 13198-13199 (2008).

42. Quek, S. Y.; Venkataraman, L.; Choi, H. J.; Louie, S. G.; Hybertsen, M. S.; Neaton, J., Amine− gold linked single-molecule circuits: experiment and theory. *Nano Lett.* **7**, 3477-3482 (2007).

43. Sun, S.; Bernstein, E., Spectroscopy of neurotransmitters and their clusters. 1. Evidence for five molecular conformers of phenethylamine in a supersonic jet expansion. *J. Am. Chem. Soc.* **118**, 5086-5095 (1996).

44. Graham, R. J.; Kroemer, R. T.; Mons, M.; Robertson, E. G.; Snoek, L. C.; Simons, J. P., Infrared ion dip spectroscopy of a noradrenaline analogue: hydrogen bonding in 2-amino-1-phenylethanol and its singly hydrated complex. *J. Phys. Chem. A*, **103**, 9706-9711 (1999).

45. Ishiuchi, S.-i.; Sone, H.; Fujii, M., Laser Desorption Supersonic Jet Spectroscopy of Octopamine by Its Hydrochloride Salt. *Chem. Lett.* **42**, 1166-1167 (2013).
